# Radiomics-based machine learning differentiates “ground-glass” opacities due to COVID-19 from acute non-COVID-19 lung disease

**DOI:** 10.1038/s41598-021-96755-0

**Published:** 2021-08-26

**Authors:** Andrea Delli Pizzi, Antonio Maria Chiarelli, Piero Chiacchiaretta, Cristina Valdesi, Pierpaolo Croce, Domenico Mastrodicasa, Michela Villani, Stefano Trebeschi, Francesco Lorenzo Serafini, Consuelo Rosa, Giulio Cocco, Riccardo Luberti, Sabrina Conte, Lucia Mazzamurro, Manuela Mereu, Rosa Lucia Patea, Valentina Panara, Stefano Marinari, Jacopo Vecchiet, Massimo Caulo

**Affiliations:** 1 Department of Neuroscience, Imaging and Clinical Sciences, “G. d’Annunzio” University, Chieti, Italy; 2 Department of Radiology, “Santissima Annunziata” Hospital, “G. d’Annunzio” University of Chieti, Via dei Vestini, 66100 Chieti, Italy; 3 Center of Advanced Studies and Technology (CAST), “G. d’Annunzio” University of Chieti-Pescara, Chieti, Italy; 4 Department of Psychological, Health and Territory Sciences, “G. d’Annunzio” University of Chieti-Pescara, Chieti, Italy; 5 Department of Radiology, Stanford University School of Medicine, Stanford, CA USA; 6 Department of Radiology, Netherlands Cancer Institute, Amsterdam, The Netherlands; 7 Department of Radiation Oncology, “Santissima Annunziata” Hospital, “G. d’Annunzio” University of Chieti, Via Dei Vestini, 66100 Chieti, Italy; 8 Unit of Ultrasound in Internal Medicine, Department of Medicine and Science of Aging, “G. D’Annunzio” University, Chieti, Italy; 9 Department of Pneumology, “Santissima Annunziata” Hospital, “G. d’Annunzio” University of Chieti, Via Dei Vestini, 66100 Chieti, Italy; 10 Clinic of Infectious Diseases, Department of Medicine and Science of Aging, University ‘G. d’Annunzio’ Chieti-Pescara, 66100 Chieti, Italy

**Keywords:** Medical imaging, Public health, Predictive markers, Infectious diseases

## Abstract

Ground-glass opacities (GGOs) are a non-specific high-resolution computed tomography (HRCT) finding tipically observed in early Coronavirus disesase 19 (COVID-19) pneumonia. However, GGOs are also seen in other acute lung diseases, thus making challenging the differential diagnosis. To this aim, we investigated the performance of a radiomics-based machine learning method to discriminate GGOs due to COVID-19 from those due to other acute lung diseases. Two sets of patients were included: a first set of 28 patients (COVID) diagnosed with COVID-19 infection confirmed by real-time polymerase chain reaction (RT-PCR) between March and April 2020 having (a) baseline HRCT at hospital admission and (b) predominant GGOs pattern on HRCT; a second set of 30 patients (nCOVID) showing (a) predominant GGOs pattern on HRCT performed between August 2019 and April 2020 and (b) availability of final diagnosis. Two readers independently segmented GGOs on HRCTs using a semi-automated approach, and radiomics features were extracted using a standard open source software (PyRadiomics). Partial least square (PLS) regression was used as the multivariate machine-learning algorithm. A leave-one-out nested cross-validation was implemented. PLS β-weights of radiomics features, including the 5% features with the largest β-weights in magnitude (top 5%), were obtained. The diagnostic performance of the radiomics model was assessed through receiver operating characteristic (ROC) analysis. The Youden’s test assessed sensitivity and specificity of the classification. A null hypothesis probability threshold of 5% was chosen (p < 0.05). The predictive model delivered an AUC of 0.868 (Youden’s index = 0.68, sensitivity = 93%, specificity 75%, p = 4.2 × 10^–7^). Of the seven features included in the top 5% features, five were texture-related. A radiomics-based machine learning signature showed the potential to accurately differentiate GGOs due to COVID-19 pneumonia from those due to other acute lung diseases. Most of the discriminant radiomics features were texture-related. This approach may assist clinician to adopt the appropriate management early, while improving the triage of patients.

## Introduction

Coronavirus disease 2019 (COVID-19) is a viral infectious disease caused by Severe Acute Respiratory Syndrome Coronavirus 2 (SARS-CoV-2), which has gradually spread worldwide since December 2019^1,2^. The clinical presentation is extremely variable and ranges from asymptomatic or paucisymptomatic infection to severe pneumonia with respiratory failure^3,4^. Typical chest computed tomography (CT) pattern presentation is bilateral and peripheral predominant ground glass opacities (GGOs) with or without superimposed septal thickening (“crazy paving” pattern), and parenchymal consolidations^5–10^. A recent metanalysis demonstrated that the most common CT findings in asymptomatic COVID-19 patients were GGOs, presenting as the only CT finding in 71% of patients^11^. Despite the reported high sensitivity of CT (94.5%), its sensitivity was relatively low (46%)^12,13^. This is partially explained to the fact that GGOs are a non-specific CT finding^7,10^. In fact, they can be found in the early, exudative phase of COVID-19 pneumonia as well as in interstitial and alveolar diseases such as pulmonary edema, alveolar hemorrhage, infectious pneumonia, hypersensitivity pneumonia, and others acute lung diseases^14–16^. For this reason, although the context of the current pandemic and the previous CT findings can be indicative of COVID-19 pneumonia, the differential diagnosis of GGOs remains a challenge^10,17^. Recently, a few studies outlined a growing interest toward imaging-based tools aimed to assist physicians in patient management^18–24^. Radiomics is one of these tools and it allows the extraction of large amounts of quantitative data from medical images^25^. A radiomics analysis includes the evaluation of the size, shape, and textural features containing spatial information on pixel or voxel intensity distribution and patterns. Radiomics features can be further integrated into machine learning models with the aim to improve diagnosis and patient management. This approach was recently investigated to improve the detection and the differential diagnosis of COVID-19 pneumonia^20,21,23,24,26–29^. For example, Zhang et al. proposed a CT-based deep learning integrated radiomics model for the differentiation of COVID-19 pneumonia from other community acquired pneumonias. They demonstrated an area under the curve (AUC) of 0.959 with a sensitivity and specificity of 0.879 and 0.887 respectively^24^. Gangloff et al., evaluated machine learning models including logistic regression, random forest and neural networks, using routine clinical and laboratory data to improve the performance of RT-PCR and chest-CT for COVID-19 diagnosis. In this way, the AUC values of chest-CT and RT-PCR was 0.892 and became 0.930 with the contribution of artificial neural network and logistic regression^20^. All the above-mentioned studies investigated the CT pattern presentation of COVID-19 considering GGOs in association with other imaging findings such as consolidation and “crazy paving”. A CT radiomics analysis of COVID-19-related GGOs and consolidation was recently proposed in a differential diagnosis with other atypical pneumonias. In this study, lesions were classified according to their skewness and the best AUC, sensitivity and specificity were 0.907, 0.830 and 0.795, respectively^21^. Since the GGOs are usually found at the early stage of COVID-19 pneumonia, we hypothesized that the early identification of their etiology, especially in patients with high clinical suspicion and negative swab, could help clinicians to promptly adopt the appropriate patient management and improve the triage activity at hospital admission.

In this study, we present a machine learning signature, based on radiomics features extracted exclusively from the GGOs on CT images, for the early differential diagnosis of GGOs due to COVID-19 pneumonia and those due to other acute lung diseases.

## Materials and methods

### Study population

**Figure 1:**
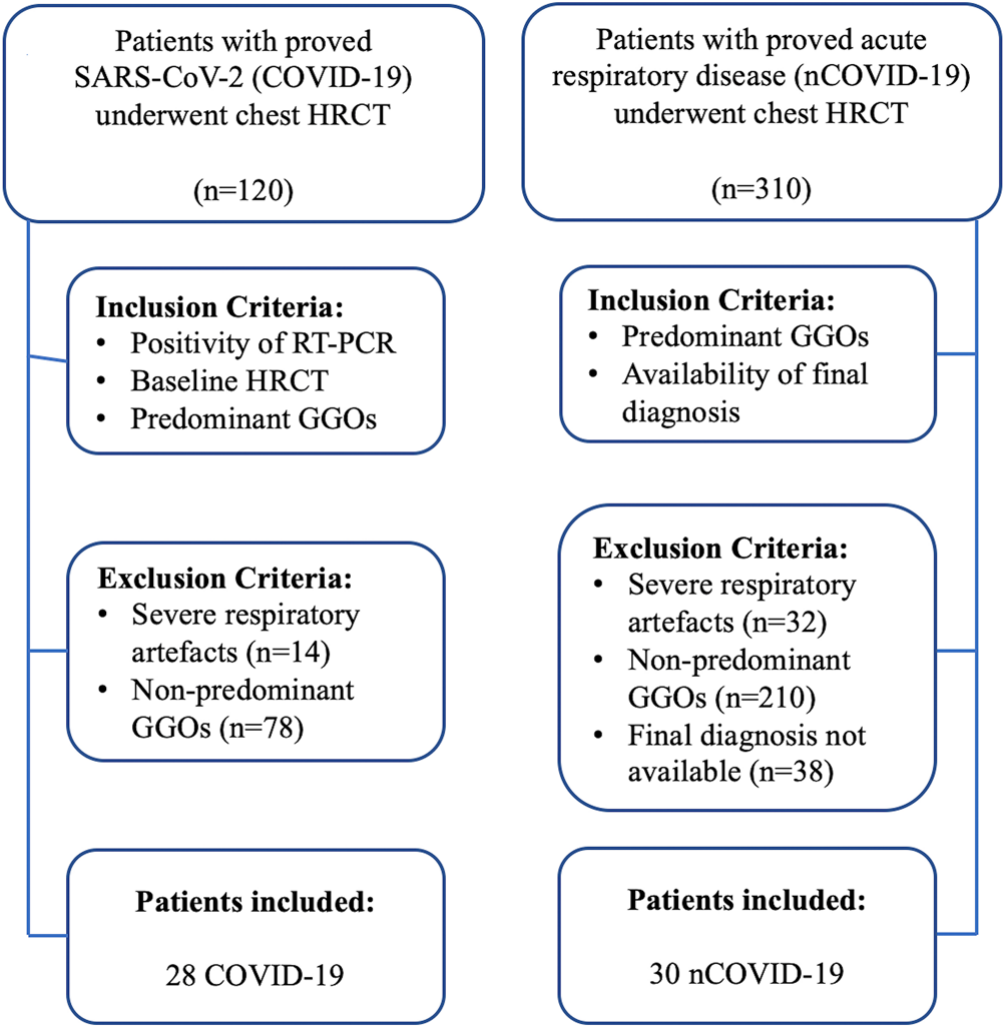
Study flowchart.

The study received formal approval from the Ethical Committee of the University G. d’Annunzio of Chieti-Pescara, Italy; informed consent was waived by the same ethics committee that approved the study (Comitato Etico per la Ricerca Biomedica delle Province di Chieti e Pescara e dell’Università degli Studi “G. d’Annunzio” di Chieti e Pescara, Italy). The study was conducted according to ethical principles laid down by the latest version of the Declaration of Helsinki. We retrospectively included a total of 120 consecutive patients diagnosed with SARS-CoV-2 infection based on RT-PCR who underwent a clinically indicated high-resolution chest CT (HRCT) between March 2020 and April 2020 at our institution. Patients were included if they met all the following criteria: (a) baseline HRCT performed at hospital admission, (b) GGO as predominant feature on chest CT scans. Another set of 310 patients (nCOVID) with clinically indicated HRCT for acute respiratory disease performed between August 2019 and April 2020 was retrospectively included in the study (nCOVID). For this second set, patients were included if they met all the following criteria: (a) GGO as predominant feature on chest CT scans, (b) availability of final diagnosis (clinical, laboratory, or pathology). The presence of a predominant GGOs pattern was assessed by two radiologists (M.M. and R.L.P.) with more than 10 years of experience in chest imaging, in consensus. More in detail, the readers assessed the presence of GGOs, consolidations, and “crazy paving” on CT images. In this regard, apart when the GGOs were the only CT finding, the predominant GGOs pattern was defined as present when the GGOs were considered like the major finding compared to consolidation and/or crazy paving pattern^10,30–33^. In the first set (COVID), we excluded 92 patients: 14 had severe respiratory artefacts, 78 had a non-predominant GGO pattern. In the second set (nCOVID) we excluded 280 patients: 32 had severe respiratory artefacts, 210 had a non-predominant GGO alteration and 38 were treated in another hospital and the final diagnosis was not available. None of the patients considered eligible for the study had a concomitant malignancy. The final study population was composed of 28 COVID and 30 nCOVID for a total of 58 patients (Fig. 1).

### CT protocol

Non-enhanced chest CT scans were performed in a supine position, during inspiratory breath-hold, from the apex to the lung bases, on a 128-slice multi-detector CT scanner (Somatom Definition AS, Siemens Healthineers, Germany). The field of view ranged between 35 and 40 cm according to the body size. The electronic window values were amplitude (W) 1200–1600 HU and window or center level (L) between − 600 and − 750 HU. The main scan parameters were: tube voltage = 120 kVp, automatic tube current modulation (30–70 mAs), pitch = 0.9–1.5 mm (0.9, 1.2 and 1.5 mm for 6, 47 and 5 patients respectively), matrix = 512 × 512. The images were reconstructed with a slice thickness of 0.625–1.250 mm (0.625, 1.000, and 1.250 mm for 46, 8, and 4 patients respectively) with the same increment with a high spatial frequency reconstruction algorithm (B50, I50).

### Image segmentation

**Figure 2:**
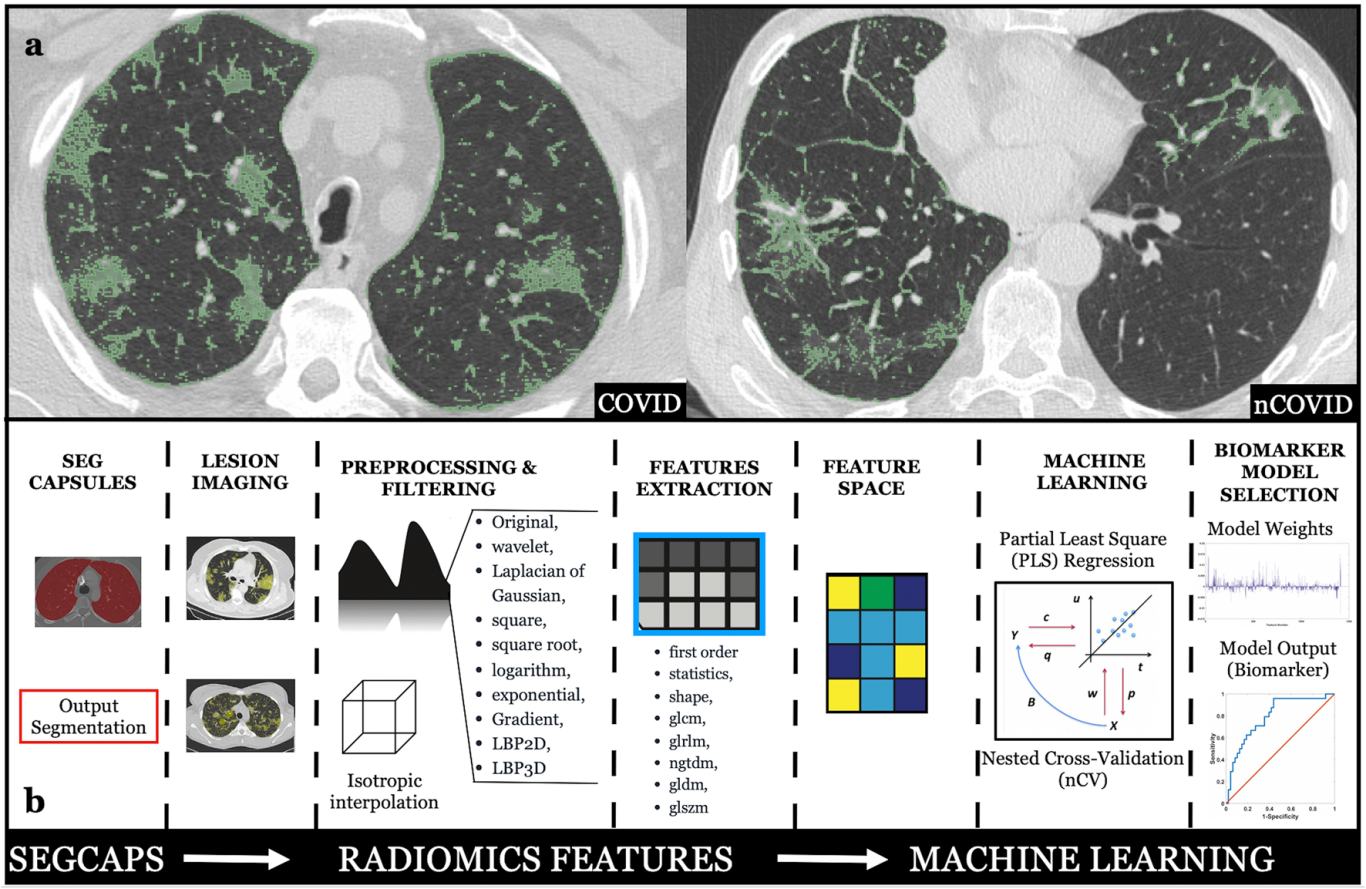
(**a**) Ground glass opacities segmentations of COVID and nCOVID on chest high-resolution computed tomography. (**b**) Schematic representation of the radiomics features extraction and the machine learning framework implemented.

A whole-volume semi-automated GGOs delineation was independently performed by two fourth year senior radiology residents (C.V. and M.V.) that were blinded from swabs results using an open-source medical image computing platform, 3DSlicer Version 4.8 (www.3dslicer.org) (Fig. 2a). In detail, the GGOs were segmented using a “threshold-effect” tool and manually setting the threshold between − 1350 and − 700 HU^8,34,35^. If necessary, the segmentation was further manually corrected by each reader in order to exclude automated segmented pixels beyond the GGOs. Once the semiautomated segmentation of GGOs was concluded, the lungs were automatically extracted via Convolutional Neural Network (CNN) algorithms to create binary mask^36^. Then, a logical “and”, between these masks and the segmentations obtained by the radiology residents, was performed (using “3dcalc”) to exclude automated segmented pixels beyond the lungs, thus obtaining the final ROIs^37^. All the ROIs were verified by a senior radiologist with more than 10 years of experience in chest imaging (M.M.) to confrirm the correct position and correspondence with the underlying CT findings.

### Radiomics analysis

The extraction of the radiomics features was conducted using PyRadiomics (https://pyradiomics.readthedocs.io), a flexible open-source platform capable of extracting a large panel of engineered features from medical images; this radiomics quantification platform enables the standardization of both feature definitions and image processing^38^. To avoid data heterogeneity bias and minimize acquisition-related radiomics variability, HRCT images were subjected to imaging resampling (2 × 2 × 2 mm) ^39^. For each ROI, ten built-in filters (Original, wavelet, Laplacian of Gaussian (LoG), square, square root, logarithm, exponential, Gradient, LBP2D, LBP3D) were applied and seven feature classes (first order statistics, shape descriptors, glcm, glrlm, ngtdm, gldm, glszm) were calculated, for a total of 1409 radiomics features. The reproducibility assessment of the features extracted from segmentations of all patients was performed.

### Machine learning approach: partial least square (PLS) regression

A machine learning (i.e. multivariate) approach was implemented to exploit radiomics features multidimensionality (Fig. 2b). Two main approaches were implemented to improve and correctly assess the generalization performance of the machine learning model^40–42^. The first approach was to reduce the number of features by selecting only those that were highly repeatable (r > 0.95) between the two masks delineated by the senior radiology residents. The second approach was to implement a machine learning framework based on a linear regression analysis that employed a space dimension reduction procedure, namely the partial least square (PLS) regression^40,43–45^. The PLS was used to differentiate COVID from nCOVID patients. Moreover, in this work, a leave-one-out nested cross-validation (nCV) was implemented to optimize the PLS number of components and to assess the PLS generalization performance^42,46–48^. The β-weights of the PLS analysis were obtained by running the algorithm on the complete dataset with the optimal number of components delivered by the nCV analysis. They linked the original independent variables with the dependent variable thus depicting the importance and sign of the original variables in the prediction. Among β-weights, top 5% features were calculated. Those features included the 5% features with the largest β-weights in magnitude thus representing the features with the highest predictive capability. The machine learning analyses were implemented in Matlab.

### Statistical analysis

The inter-reader correlation of radiomics features was assessed using an across-subjects Person correlation coefficient. Only radiomics features with high correlation coefficient (above 0.95) were used within the machine learning model^49^. The COVID vs nCOVID classification performance was assessed through Receiver Operating Characteristic (ROC) analysis comparing the inferred (out-of-training-sample) with the true group. COVID patients were attributed to the “positive” group, whereas nCOVID patients were attributed to the “negative” group. The ROC analysis was also performed on random shuffled group labels to simulate the null hypothesis and evaluate its confidence interval (repeated 10^6^ times). The ROC analysis delivered an Area Under the Curve (AUC), which could be transformed into a z-score for assessing its statistical significance by using the random shuffled group labels. The Youden’s test was used to calculate the sensitivity and specificity of the ROC analysis. 5% null hypothesis probability threshold was chosen (p < 0.05). The statistical analysis was performed in MATLAB.

### Ethical statement

This study was approved by the local ethics committee. The study used only pre-existing medical data, therefore patient consent was waived.

## Results

### Study population

The majority of patients included in the study were male (n = 34, 59%), and the median age was 66 years (interquartile range 55–81). Out of the total patient population (n = 58), 28 (48%) were assigned to the COVID group, and 30 (52%) to the nCOVID group. The nCOVID group (n = 30) included four with Cytomegalovirus (CMV) pneumonia, two with pulmonary edema, five with Acute Respiratory Distress Syndrome (ARDS), eight with Organizing Pneumonia, three with Pneumocystis Jirovecii pneumonia, two with Influenza A pneumonia, two with Legionella pneumonia, three with alveolar hemorrhage and one with hypersensitivity pneumonia (Table 1).

**Table 1:** Descriptive baseline characteristics of our study population (n = 58).

Variable	Value (%)
**Gender**	
Male	34/58 (59%)
Female	24/58 (41%)
Median age (IQR)	66 (55–81)
HRCT exam	58
**nCOVID group (n = 30)**	
Citomegalovirus pneumonia	4 (6.9%)
Pulmonary edema	2 (3.4%)
Acute distress respiratory syndrome	5 (8.6%)
Organizing pneumonia	8 (13.8%)
Alveolar hemorrage	3 (5.2%)
Hypersensitivity pneumonia	1 (1.7%)
Influenza A pneumonia	2 (3.4%)
Legionella pneumonia	2 (3.4%)
Pneumocysisi jirovecii pneumonia	3 (5.2%)
**COVID group (n = 28)**	
COVID-19 pneumonia	28 (48.4%)

*IQR* Inter-Quartile Range, *HRCT* High-Resolution CT.

### Radiomics-based machine learning

**Figure 3:**
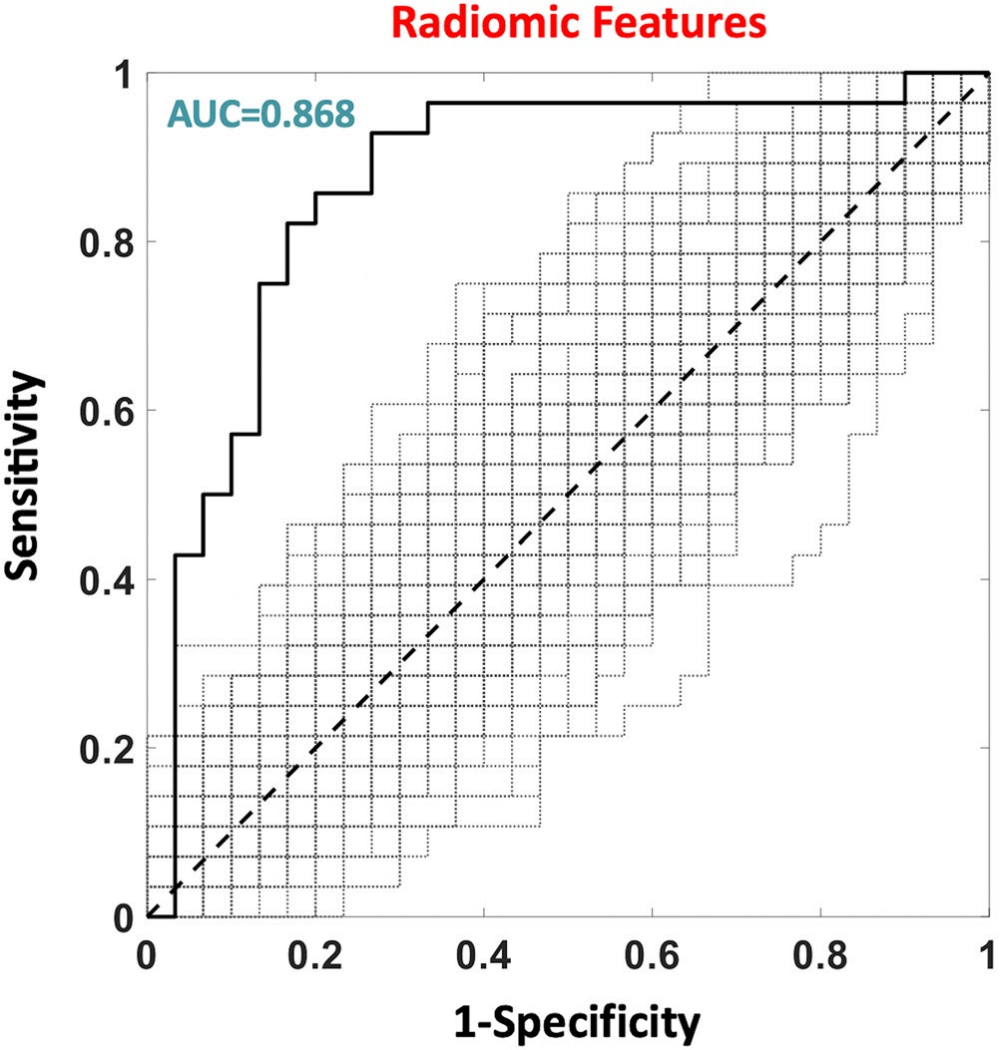
ROC analysis of the machine learning (PLS) classification performance (COVID patients were labelled as “positive”, nCOVID patients as “negative”).

**Figure 4:**
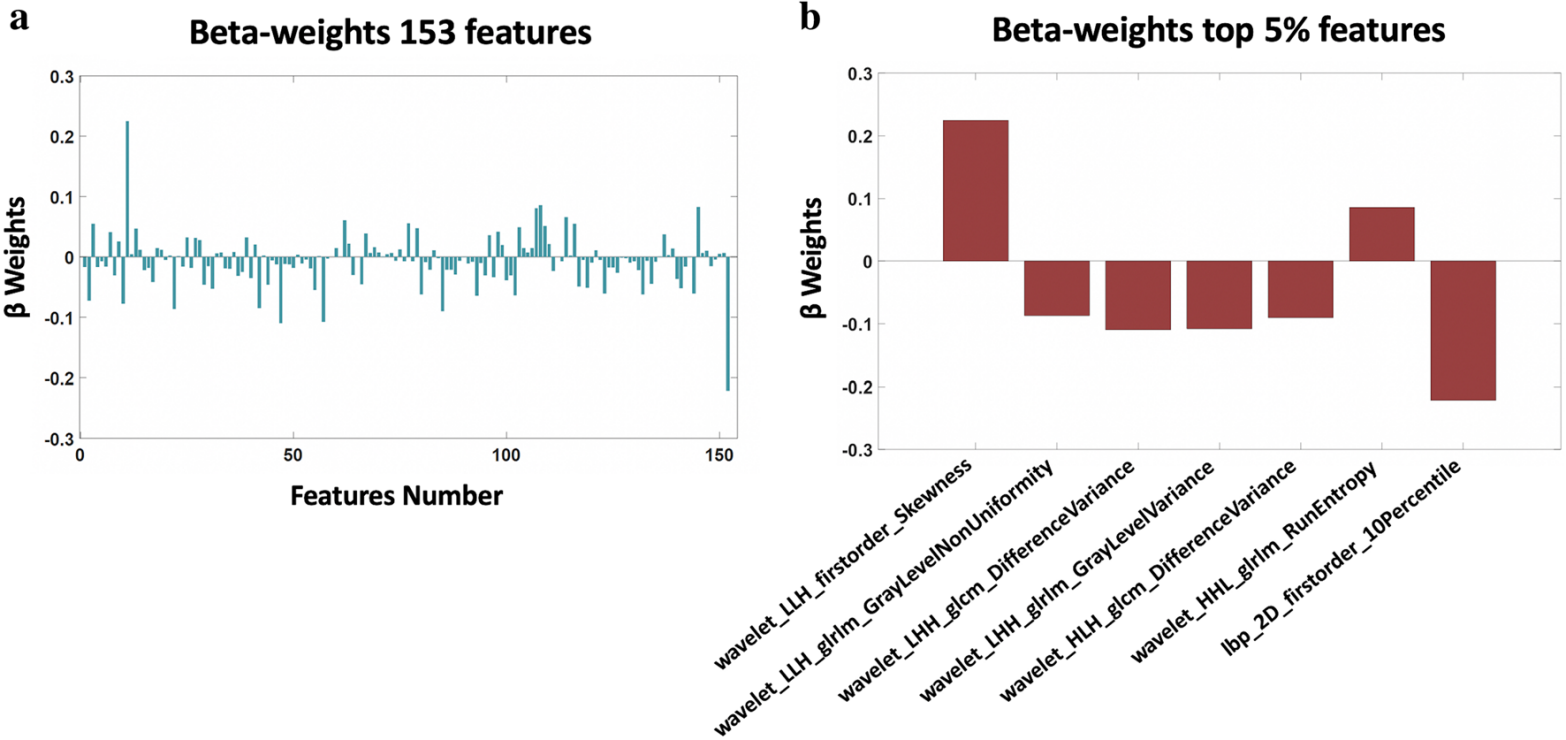
(**a)** Partial least square β-weights associated with reliable (r > 0.95) radiomics features. (**b**) β-Weights associated to the top 5% of features with the largest β-weights in magnitude.

A total of 1409 radiomics features were extracted. 153 of these features showed an inter-reader correlation of r > 0.95 and were used for further analysis. When employing radiomics features with an r > 0.95, i.e., 153 radiomics features, an AUC = 0.868 was obtained (z = 5.1, p = 4.2 × 10^–7^, Fig. 3). A Youden’s index of 0.68 was associated with a sensitivity and specificity of 93% and 75% respectively (Table 2). The estimated optimal number of PLS components, evaluated within the nCV framework, was 7. The weights of the PLS (β-weights) are shown in (Fig. 4a,b). Since a value of “1” was attributed to the COVID patients and a value of “0” was attributed to the nCOVID patients during the machine learning training, a positive weight suggests a higher feature value in COVID compared to nCOVID patients with an opposite behavior for a negative weight. Of the top 5% features, 5 (wavelet_LLH_glrlm_GrayLevelNonUniformity, wavelet_LHH_glcm_DifferenceVariance, wavelet_LHH_glrlm_GrayLevelVariance, wavelet_HLH_glcm_DifferenceVariance, wavelet_HHL_glrlm_RunEntropy), were associated to glrlm and glcm texture matrices (second second order features), and 2 (wavelet_LLH_firstorder_Skewness, Ibp_2D_firstorder_10Percentile) were related to the image intensity distribution (first order features). All, except one, second order features had a negative weight, meaning that COVID-19 patients (labelled as 1 in the classification algorithm) tended to have a more homogeneous texture. Of the two first order features, one had positive weight (the skweness, larger value in COVID-19 patients) and one had a negative weight (the 10th percentile, smaller value in COVID-19 patients) indicating that COVID group, although having a distribution of image intensities with average equal values that nCOVID group, had a larger occurrence of low intensity pixels.

**Table 2:** Diagnostic performance of the radiomics-based machine learning signature including area under the curve (AUC), Youden’s index, sensitivity, specificity and p-value.

Area under the curve (AUC)	Youden’s index^a^	Sensitivity^a^	Specificity^a^	p-value	Top 5% features^b^	β-Weights
0.868	0.68	0.930	0.750	4.2 × 10^–7^	wavelet_LLH_glrlm_GrayLevelNonUniformity	− 0.08
					wavelet_LHH_glcm_DifferenceVariance	− 0.12
					wavelet_LHH_glrlm_GrayLevelVariance	− 0.11
					wavelet_HLH_glcm_DifferenceVariance	− 0.09
					wavelet_HHL_glrlm_RunEntropy	0.07
					wavelet_LLH_firstorder_Skewness	0.23
					Ibp_2D_firstorder_10Percentile	− 0.23

In the last two columns on the right, the 5% features with the largest β-weights in magnitude (top 5% features) and their β-weights.

^a^Youden’s test.

^b^5% features with the largest β-weights in magnitude in Partial Least Square analysis.

## Discussion

Our results demonstrated that a machine learning signature based on radiomics features extracted from GGOs on CT images is an accurate method to early differentiate COVID-19 pneumonia from other acute non-COVID-19 lung diseases. These results confirm the promising role of radiomics in the diagnosis of COVID-19 pneumonia and are in line with the most recent literature on this topic^21–24^. For example, Huang et al. used radiomics to discriminate COVID-19 and influenza pneumonia by combining CT signs and quantitative features extracted from the initial unenhanced CT images^22^. The resulting AUC was 0.959 and it was associated with 89.9% sensitivity and 90.7% specificity. On the other hand, Gulbay et al. focused on radiomics-based features extracted from GGOs and consolidation to differentiate COVID-19 and other atypical pneumonia. They classified the lesions according their skewness and the group-specific models that were creaded showed an AUC ranging between 0.774 and 0.907.

Of note, none of the above-mentioned studies was specifically focused on GGOs and other previous studies investigating the differential diagnosis of GGOs were conducted in pre-COVID-19 era^50,51^. In this regard, overcoming the limited specificity of GGOs on CT images assumes even more relevance in the pandemic scenario since they represent the most common CT findings in the early phase of COVID-19 pneumonia^10^. In fact, the early identification of the GGOs etiology could help to promptly adopt the appropriate management and reduce the burden on the emergency department. For instance, patients admitted to the hospital for suspected COVID-19 pneumonia are temporarily placed in dedicated COVID-19 rule-out units, and they may experience a delay in care or intervention^10^. In this scenario, chest CT is used as a surrogate for the early identification of COVID-19 pneumonia and may help the triage activity by identifying an alternative diagnosis and by improving the patient selection for intensive/non-intensive care in case of clinical worsening. Furthermore, the treatment of GGOs varies according to their etiology. For example, patients with organizing pneumonia are usually treated with corticosteroid therapy with the occasional addition of antibiotics^52^. On the other hand, corticosteroids are recommended only in patients with severe and critical COVID-19 infection^53^.

Interestingly, five of the seven features included in the top 5% predictive features of our study were texture related, thus indicating that the lesion hetereogeneity may help the differential diagnosis of GGOs. These results are in line with recent studies on the differential diagnosis of COVID-19 pneumonia^21,54,55^. For instance, Gulbay et al. showed that the mean skewness and texture features were significantly different in GGOs when comparing COVID-19 and atypical pneumonia^21^. Moreover, four out of five texure-related features included in our model revealed a higher homogeneity in GGOs of COVID-19 than in those of non-COVID-19 patients. We speculated that the higher homogeneity in COVID-19 pneumonia may reflect the degree of inflammatory infiltrate in the early stage of diffuse alveolar damage. In fact, GGOs are typically observed in the exudative phase of COVID-19 pneumonia, which is characterized by interstitial and alveolar oedema, hemorrhage, and hyaline membrane formation. With the progression of the disease, GGOs increase in density and heterogeneity, thus evolving in a more consolidative pattern or with a “crazy paving” pattern^16^.

Although our results are promising, there are some limitations. First, our study included a relatively low number of patients. Nonetheless, our investigation was intended as a proof-of-concept study and, since our aim was focused on GGOs, our inclusion criteria were necessarily strict, considering only patients with predominant GGOs pattern. Moreover, GGOs are typically found in the acute phase of the disease, which may not correspond with the timing of CT and this may have further reduced the study population. Second, compared to the number of patients included in the study, we analyzed a large number of predictive features. In this regard, the PLS exploited the high collinearity of the different radiomics features, thus delivering a high prediction performance. Moreover, the cross-validation modality delivered an evaluation of the out-of-training sample performance using the sample numerosity available that is unbiased. Hence, we expect that by reducing the ratio between features and subject, the model prediction may further increase. Third, this is a retrospectively designed, single-center study. Further prospective and possibly multicentric studies are warranted to define a more standardized approach.

## Conclusion

A radiomics-based machine learning signature showed the potential to accurately differentiate GGOs due to COVID-19 pneumonia from those due to other acute lung diseases on HRCT scans. Most of the discriminant radiomics features were related to the texture analysis. After a careful prospective evaluation in larger multicentric studies, this approach may assist clinicians to adopt the appropriate management early, while improving the triage of patients.

## Data Availability

The datasets generated during and/or analyzed during the current study are not publicly available due to the clinical and confidential nature of the material but can be made available from the corresponding author on reasonable request.

## Abbreviations

AUC: Area under the curve
ARDS: Acute Respiratory Distress Syndrome
CMV: Citomegalovirus
CNN: Convolutional Neural Network
COVID-19: Coronavirus disease 2019
GGO: Ground glass opacity
HRCT: High-resolution computed tomography
HU: Hounsfield unit
kvP: Kilovoltage peak
LoG: Laplacian of Gaussian
PLS: Partial least square
ROC: Receiver operating characteristic curve
ROI: Region of interest
RT-PCR: Real-time polymerase chain reaction
SARS-CoV-2: Severe Acute Respiratory Syndrome Coronavirus 2
